# Metabolome and microbiome profiling of a stress-sensitive rat model of gut-brain axis dysfunction

**DOI:** 10.1038/s41598-019-50593-3

**Published:** 2019-10-01

**Authors:** Shalome A. Bassett, Wayne Young, Karl Fraser, Julie E. Dalziel, Jim Webster, Leigh Ryan, Patrick Fitzgerald, Catherine Stanton, Timothy G. Dinan, John F. Cryan, Gerard Clarke, Niall Hyland, Nicole C. Roy

**Affiliations:** 1 Food Nutrition & Health, AgResearch Ltd., Grasslands Research Centre, Tennent Drive, Palmerston North, 4442 New Zealand; 2 Riddet Institute, Massey University, Palmerston North, New Zealand; 3 High-Value Nutrition National Science Challenge, Auckland, New Zealand; 4 Farm Systems North, AgResearch Ltd., Ruakura Research Centre, Hamilton, New Zealand; 5 Laboratory of Neurogastroenterology, APC Microbiome Ireland, University College Cork, Cork, Ireland; 6 Department of Anatomy and Neuroscience, University College Cork, Cork, Ireland; 7 Department of Psychiatry and Neurobehavioural Science, University College Cork, Cork, Ireland; 8 Teagasc Food Research Centre, Moorepark, Fermoy, Co. Cork Ireland; 9 Department of Physiology, University College Cork, Cork, Ireland

**Keywords:** Microbiome, Stress and resilience, Fat metabolism

## Abstract

Stress negatively impacts gut and brain health. Individual differences in response to stress have been linked to genetic and environmental factors and more recently, a role for the gut microbiota in the regulation of stress-related changes has been demonstrated. However, the mechanisms by which these factors influence each other are poorly understood, and there are currently no established robust biomarkers of stress susceptibility. To determine the metabolic and microbial signatures underpinning physiological stress responses, we compared stress-sensitive Wistar Kyoto (WKY) rats to the normo-anxious Sprague Dawley (SD) strain. Here we report that acute stress-induced strain-specific changes in brain lipid metabolites were a prominent feature in WKY rats. The relative abundance of *Lactococcus* correlated with the relative proportions of many brain lipids. In contrast, plasma lipids were significantly elevated in response to stress in SD rats, but not in WKY rats. Supporting these findings, we found that the greatest difference between the SD and WKY microbiomes were the predicted relative abundance of microbial genes involved in lipid and energy metabolism. Our results provide potential insights for developing novel biomarkers of stress vulnerability, some of which appear genotype specific.

## Introduction

The microbiome-gut-brain axis is influenced by stress while the gut microbiota plays a significant role in regulating stress-related responses^1^. Given the significant comorbidity of stress-related disorders and gut disorders, animal models of stress and anxiety are routinely used to study functional gastrointestinal disorders such as irritable bowel syndrome (IBS)^2–5^ as well as to develop new, more efficient pharmacological and/or behavioural treatments^2,6–8^. More recently, these models have also been applied to study the effects of diet and food components on stress^9–11^. However, the physiological factors that underlie stress sensitivity are not well understood.

The idea that psychological wellbeing is influenced by the gut microbiota is gaining momentum^12–14^. However, because communication between the gut and its microbiome and the brain is bidirectional, the origin of the causative factors in stress susceptibility involving this axis remain elusive. Stress can trigger anxiety and depression^15,16^ and is a significant risk factor for IBS and the associated symptoms^17^. Chronic stress can also alter microbiota composition^18,19^, and compositional alterations of the gut microbiota has been associated with both IBS and depression^20^. Of note, recent evidence has also suggested that, in the majority of patients, IBS and functional dyspepsia originate in the gut and precede the onset of anxiety and depression^21^. Yet whether or not fluctuations in gut microbiota composition and stability is the consequence or cause of these adverse health states remains unclear.

The Wistar Kyoto (WKY) rat strain is a model commonly used to study stress, anxiety and depression, as well as visceral hypersensitivity^22,23^ general. WKY rats have a greater innate sensitive to stress and display visceral hypersensitivity than Sprague-Dawley (SD) rats^24^. As such, these rat strains have also been used as models for studying gut-brain axis (GBA) dysfunction^25^ because susceptibility to chronic stress often results in anxiety and gut microbiota alterations^26^.

Little is known about the metabolic changes associated with stress in either WKY or SD rats. Understanding such changes may be important for better understanding stress in humans, where the search for reliable biomarkers to assist with accurate diagnosis, prevention and treatment of stress-related mental illnesses remains on-going^27^. Untargeted metabolomics using MS-based methods can provide a global snapshot of the metabolites present in a biological system, a key early step in identifying new potential biomarkers and impacted pathways^28^. This discovery-based approach has recently been used to identify metabolic changes in plasma thought to be involved in stress-related diseases, such as major depressive disorders^29,30^. These results suggest that, in humans, depression may be associated with alterations in the metabolism of lipids, amino acids and neurotransmitters^29,30^. However, whether this is also the case in rodent models is currently unknown. There is also limited knowledge of which microbial signatures underpin physiological responses to stress in these rat strains, or the interrelationship between these. Understanding the association between acute stress and the microbiota in WKY and SD animals will help to define an appropriate response phenotype and provide insights into dysregulated host-microbiota physiology, which may be relevant for human stress-related conditions.

In this study, we hypothesized that factors influencing the gut-brain axis (GBA), such as host metabolism, play a key role in the susceptibility to stress-related disorders. We investigated the functional response to acute stress in WKY and SD rats by examining the stress-induced effect of the forced swim test on metabolic activity and microbiota composition. Stress-induced alterations in metabolites and caecal microbiota were identified which could serve as potential biomarkers for studying the effect of dietary or pharmacological treatments on behaviour, stress, and anxiety, in addition to supporting the selection of appropriate rodent models for future work. These biomarkers may also provide further insight regarding the pathophysiology of human depression and the effectiveness of novel therapies.

## Results

### Open field test (OFT)

WKY rats displayed a significantly lower velocity (*P* < 0.001), travelled a shorter distance (*P* < 0.001), entered the inner zone of the arena less (*P* < 0.01) and spent less time in the inner zone (*P* < 0.01) than SD rats as shown in Supplementary Fig. S1.

### Novel object recognition (NOR)

Both strains spent more time exploring the novel rather than familiar object as shown by the total exploration time and discrimination index. There was no significant difference between strains (Supplementary Fig. S2).

### Forced swim test (FST)

The FST-induced stress response was used to verify that the WKY rats displayed an acute stress hypothalamic pituitary adrenal axis (HPA) response and accompanying behaviour profile and physiology. It also provided an acute stress challenge to the function of the microbiome. SD rats spent more time swimming (*P* = 0.005) and climbing (*P* < 0.001) than WKY rats (Supplementary Fig. S3). WKY rats spent more time in an immobile state than SD rats (*P* < 0.001). SD rats produced more faecal pellets than WKY rats during the FST (*P* < 0.001).

### Stress-induced corticosterone levels

Corticosterone levels increased in response to acute stress (both strains; *P* < 0.001), as measured by pre-FST levels compared to postmortem levels, but there was no difference between rat strains (*P* = 0.55) as shown in Supplementary Table S1. Corticosterone levels were also higher in stressed than non-stressed (control) rats (both strains; *P* < 0.001) but there was no difference between rat strains (*P* = 0.87).

This result confirmed that the animals were stressed by the FST.

### LC-MS metabolomic profiling of plasma and brain

Table 1 shows the number of features for polar and lipid metabolites obtained in both positive- and negative-ion modes used for statistical analysis, along with the number of components identified (listed in brackets). Positive and negative ion data for each biological sample and analysis mode were combined resulting in four data sets for multivariate analysis. Principle component analysis (PCA) showed the run-order correction process was successful and the data were of suitable quality for further statistical analysis. PCA revealed only minor run order effects in a few analyses which were corrected using a linear regression function in R. The quality controls for all analyses demonstrated the data collected were suitable for further analysis.

**Table 1:** Number of features (and annotated components) from each analytical stream used for statistical analysis.

Analysis mode	Plasma	Brain
Polar positive	657 (22)	496 (22)
Polar negative	552 (16)	310 (21)
Lipid positive	1017 (143)	624 (166)
Lipid negative	821 (40)	621 (96)

### Brain and plasma polar metabolites

**Figure 1:**
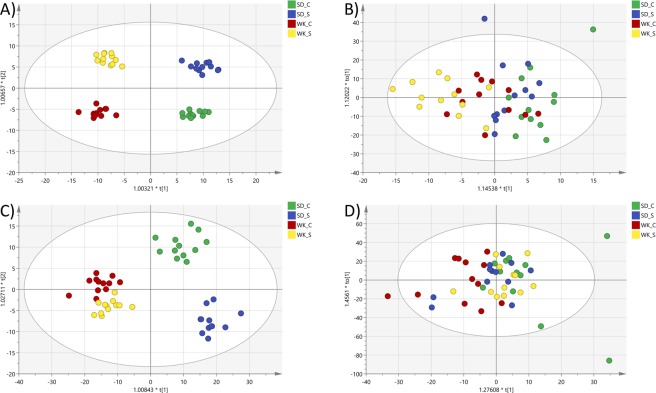
Score plots of OPLS-DA models for plasma (**A**) and brain (**B**) polar metabolites, and plasma (**C**) and brain (**D**) lipids. SD_C = Sprague Dawley (Control), SD_S = Sprague Dawley (Stressed), WK_C = Wistar Kyoto (Control), WK_S = Wistar Kyoto (Stressed).

Orthogonal partial least squares discriminant analysis (OPLS-DA) showed metabolites profiles were altered by stress. The OPLS-DA model for plasma (Fig. 1A) was significant (*P* = 0.0005) with R^2^(Y) and Q^2^(cum) values of 96.9% and 48.8%, respectively. However, strain differences were not significantly resolved by this model for the brain polar metabolites (Fig. 1B) and, because stress was the focus of the data analysis, data for both plasma and brain was separated by rat strain and reanalysed using univariate tools.

Brain and plasma lipid data were examined at the lipid class level by summing all lipid species of the same class within the respective sample matrix and performing univariate analysis. No significant differences in either brain or plasma were observed between any lipid class due to stress (data not shown).

In the brain, there was no significant difference in polar metabolites between stressed and control rats of either strain after false discovery rate (FDR) correction (Table 2). We did note some evidence that glutamine and tyrosine were elevated and DHA reduced in stressed SD rat brain (*P* < 0.01; FDR < 0.5) in both ionization modes. Creatine was increased in brain of both rat strains in response to stress.

**Table 2:** Brain metabolites significantly differing in abundance between stressed and control animals. **S** = stressed; **C** = control; **FDR** = false discovery rate; where FDR < 0.1 for HILIC (polar) and FDR < 0.05 for lipid analysis were considered significant.

Rat Stain	Metabolite		*P*-value	FDR	Log_2_FC (T/NT)
**Sprague Dawley**	**Polar**	Creatine	0.017	0.682	1.7732
		L-glutamine	0.008	0.457	0.1753
		Tyrosine	0.006	0.457	0.2034
		DHA (fragment)	0.009	0.457	−0.4265
**Wistar-Kyoto**	**Polar**	Creatine	0.0005	0.222	0.288
	**Lipid**	SM(d34:1)	0.021	0.063	0.267
		SM(d36:1)	0.004	0.063	0.015
		SM(d38:1)	0.001	0.063	0.122
		SM(d40:0)	0.058	0.241	0.258
		SM(d42:0)	0.019	0.241	0.019
		SM(d42:2)	0.018	0.063	0.041
		CerG1(d40:2)	0.017	0.241	−0.060
		CerG1(d41:0)	0.016	0.241	0.113
		CerG1(d42:2)	0.017	0.241	−0.100
		LPE(16:0)	0.076	0.122	−0.233
		LPE(20:1)	0.084	0.132	−0.072
		LPC(18:0)	0.016	0.241	−0.335
		DG(34:1)	0.036	0.241	−0.038
		DG(36:1)	0.020	0.241	−0.053
		DG(36:2)	0.061	0.241	−0.118
		DG(38:6)	0.017	0.241	−0.084
		PI(40:6)	0.047	0.241	0.318
		PE(34:1)	0.018	0.063	−0.128
		PE(36:1)	0.015	0.063	−0.178
		PE(36:2)	0.008	0.063	−0.033
		PE(36:3)	0.003	0.063	−0.253
		PE(38:1)	0.011	0.063	−0.171
		PE(38:2)	0.018	0.063	−0.162
		PE(38:5)	0.028	0.067	−0.165
		PE(38:6)	0.017	0.063	−0.152
		PE(39:0)	0.019	0.063	−0.045
		PE(40:6)	0.032	0.072	−0.198
		PE(40:8)	0.041	0.083	−0.678
		PE(42:1)	0.011	0.063	−0.029
		PE(42:2)	0.018	0.063	−0.006
		PE(42:10)	0.018	0.063	−0.242
		PE(44:1)	0.012	0.063	−0.029
		PE(44:2)	0.019	0.063	−0.075
		PE(44:10)	0.012	0.063	−0.116
		PS(36:1)	0.034	0.075	−0.126
		PS(36:2)	0.020	0.063	−0.126
		PS(38:1)	0.030	0.068	−0.010
		PS(38:3)	0.035	0.075	−0.123
		PS(40:2)	0.035	0.075	−0.056
		PS(41:5)	0.017	0.063	−0.245
		PS(43:5)	0.012	0.063	−0.169
		PS(43:6)	0.012	0.063	−0.159
		PS(44:10)	0.023	0.063	−0.116
		PC(38:1)	0.055	0.241	−0.091
		PC(40:2)	0.049	0.241	−0.045
		PC(42:2)	0.064	0.241	−0.093
		PC(42:7)	0.032	0.241	−0.127
		PC(44:2)	0.058	0.241	−0.063
		PC(44:12)	0.022	0.241	−0.069

Fold change and significance values of differential polar metabolites in the plasma of SD and WKY rats in response to stress are shown in Table 3. For WKY rats, glutamic acid, gamma-aminobutyric acid (GABA), and 3-methoxytyrosine were significantly lower in the plasma of stressed animals. These metabolites also trended lower in the stressed SD rats. As observed in the brain, tyrosine again trended to higher levels in stressed SD rats. Cytosine was decreased, and methionine trended toward an increase in stressed WKY rats.

**Table 3:** Plasma metabolites significantly differing in abundance between stressed and control animals.

Rat strain	Metabolite		*P*-value	FDR	Log_2_ FC (S/C)
**Sprague Dawley**	**Polar**	Glutamic acid	0.0045	0.245	−0.710
		3-Methoxytyrosine	0.0004	0.185	−0.674
		Tyrosine	0.003	0.185	0.450
		GABA	0.001	0.206	−0.777
	**Lipids**	TG(62:4)	0.0008	0.101	1.077
		TG(58:1)	0.001	0.101	1.203
		TG(60:2)	0.0015	0.101	1.194
		TG(55:0)	0.0019	0.108	1.039
		TG(59:2)	0.0025	0.108	1.178
		TG(64:3)	0.0028	0.108	1.092
		TG(57:1)	0.0028	0.108	1.181
		TG(60:4)	0.0039	0.141	0.900
		Palmitic acid	0.0004	0.231	0.673
		Vaccenic acid	0.0012	0.231	0.850
		Linoleic acid	0.0017	0.231	0.781
**Wistar-Kyoto**	**Polar**	Glutamic acid	1.94e-4	0.0191	−0.861
		3-Methoxytyrosine	1.86e-7	9.17e-5	−0.870
		GABA	0.0004	0.032	−0.801
		Cytosine	0.0023	0.096	−0.264
		Methionine DL-	0.0032	0.105	0.159

S = stressed; C = control; FDR = false discovery rate; where FDR < 0.1 for HILIC (polar) and FDR < 0.05 for lipid analysis were considered significant.

Differences between the strains (control groups) in the plasma polar metabolites were detected by supervised statistical approaches and the metabolites responsible have been reported elsewhere^31^.

### Brain and plasma lipidomics

Rat strain differences were not significantly resolved for brain lipids by the OPLS-DA model (Fig. 1D). Consequently, data analysis was performed within rat strains for the effect of stress using univariate tools. WKY rats had 49 different brain lipid species between non-stressed (control) and stressed groups that reached close to significance following FDR correction. This was not observed for SD rats (Table 2). For example, while some phosphatidylethanolamine (PE) were not significant, as a whole group they consistently shifted negatively. In particular, sphingomyelins (SM) and phosphatidylinositol (PI) were trended toward an increase in the stressed WKY rat brain. Along with PE, groupings of phosphatidylserine (PS), phosphatidylcholine (PC), lysophosphatidylcholine (LPC), lysophosphatidylethanolamine (LPE) and diacylglycerol (DG), also trended towards a decrease. Of the glycosphingolipids (CerG1) that were annotated, one trended toward an increase in response to stress while two showed a decreasing trend.

In contrast, no significant difference in plasma lipids were detected in WKY rats when stressed, yet a range of plasma triacylglycerides (TAGs) were trended higher in SD rats. Free fatty acids (vaccenic, linoleic and palmitic) were also increased in response to stress in SD rats (Table 3). Of the 87 circulating triacylglycerides annotated in plasma, 57 were increased by > 30% (which is generally considered to be physiologically relevant) in SD rats in response to stress compared to the SD control rats (Supplementary Table S3), whereas only two were decreased by more than 30%.

Differences in plasma lipids between rat strains (non-tested control groups) were detected (Fig. 1C) using the OPLS-DA model (*P* = 0.0001) with R^2^(Y) and Q^2^(cum) values of 62.0% and 32.9%, respectively.

### Caecal microbiota and short chain fatty acids (SCFAs)

**Figure 2:**
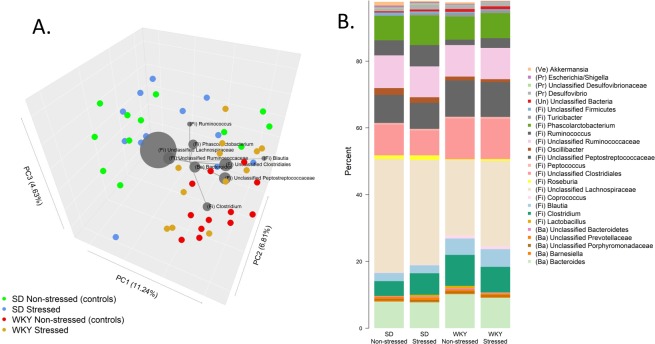
Caecal Microbiota. (**A**) PCoA biplot of unweighted Unifrac phylogenetic distances of the caecal communities in SD and WKY rats. Grey circles show the nine most relatively abundant genera where diameter is proportional to the mean relative abundance across all samples, with distance from origin (X0, Y0, Z0) indicating contribution to the variation along the principal components. (**B**) Bar plot of the mean relative abundance of the 25 most prevalent bacterial genera across all samples. Codes in parentheses indicate phylum; Ve = Verrucomicrobia, Pr = Proteobacteria, Fi = Firmicutes, Ba = Bacteroidetes, Un = Unclassified.

Gut microbiota communities of SD and WKY rats could be differentiated by principle coordinate analysis (PCoA) of unweighted Unifrac phylogenetic distances (Fig. 2A) and taxonomic composition (Fig. 2B); these included *Ruminococcus* (SD 5.45 ± 0.68; WKY 2.25 ± 0.35; % ± SEM; *FDR* < 0.001), *Blautia* (SD 2.4 ± 0.42; WKY 5.11 ± 0.75; % ± SEM; *FDR* = 0.004), and unclassified *Lachnospiraceae* (SD 32.6 ± 2.34; WKY 24.01 ± 1.36; % ± SEM; *FDR* = 0.004). However, the genera were differentially altered at the end of the FST between SD and WKY rats. Relative abundances of *Desulfovibrio*, unclassified *Desulfovibrionales* and unclassified *Alphaproteobacteria* (all of which belong to the *Proteobacteria* phylum) were increased in stressed SD rats compared to controls (*P* < 0.02), although after multiple testing adjustment the difference was not significant (FDR = 0.8). Furthermore, these taxa made up only a relatively small proportion of the microbiota; collectively they accounted for 1% of the community in SD rats and 1.75% in WKY rats. Following the FST, there were altered proportions of some *Proteobacteria* in SD rats, whereas in WKY rats, proportions of several taxa belonging to the *Firmicutes* phylum were altered. Changes in WKY rats post-FST included increased proportions of *Ruminococcus* (WKY stressed 2.92%; WKY controls 1.6%; *P* = 0.05), *Roseburia* (WKY stressed 0.6%; WKY controls 0.3%; *P* = 0.03), and *Lactococcus* (WKY stressed 0.09%; WKY controls 0.03%; *P* < 0.001), and decreased proportions of *Lactobacillus* (WKY stressed 0.19%; WKY controls 0.70%; *P* = 0.02). However, after FDR adjustment, only the change in *Lactococcus* proportions was significant (FDR < 0.001). Although only a limited number of low relative abundance taxa appeared to shift after exposure to the experimental procedures, an overall pattern could be discerned in WKY rats using random forest classification (Supplementary Table S2), where 10/12 control WKY rats were correctly classified as control, and 10/12 stressed WKY rats were correctly classified (overall error rate = 16.7%). The taxa that contributed the most to the random forest decision trees were the genera *Rothia* from the *Actinobacteria* phylum, and *Lactococcus*, *Allobaculum*, and *Anaerosporobacter*, all of which belong to the *Firmicutes* phylum. In contrast, random forest classification was unable to discern tested SD rats from non-tested SD rats, with an overall classification error rate of 58%.

Analysis of the core microbiome identified 21 taxa up to the genus level that were present in all rats; these included *Rothia*, *Bacteroides*, *Prevotella*, *Lactococcus*, *Clostridium*, *Blautia*, *Roseburia*, and *Desulfovibrio*. Further analysis showed 46 genus level taxa in > 75% of the rats. Overall, 99 genus level taxa were identified across all rats.

**Figure 3:**
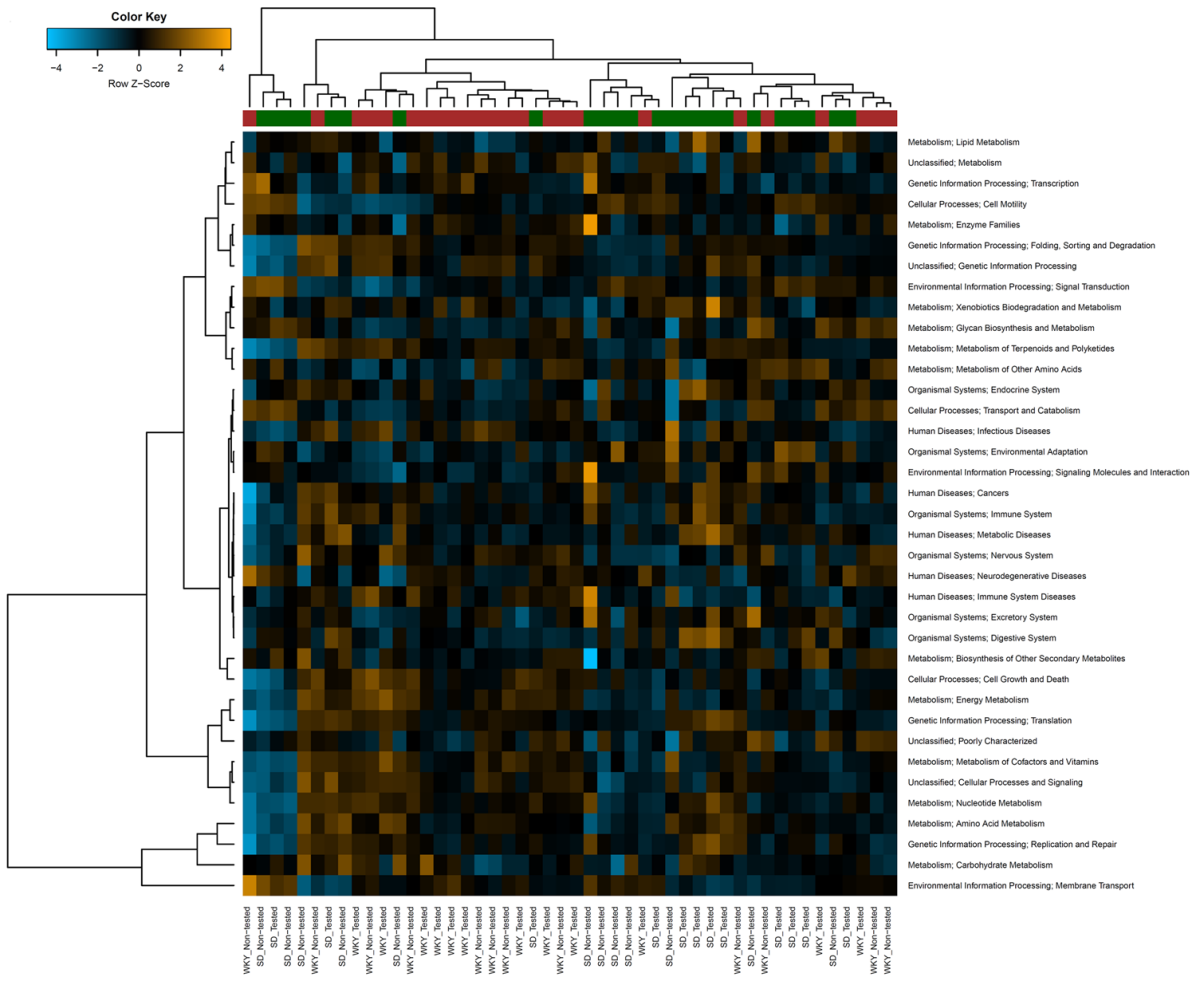
Heat map showing hierarchical clustering of predicted metagenome KEGG level 2 functions in the caecal microbiome of SD and WKY rats exposed to acute stress or non-stressed controls. The colour ribbon beneath the upper dendrogram indicates rat strain; WKY (red), SD (green).

PICRUSt analysis of the predicted bacterial metagenomes, based on the 16S rRNA analysis, showed the microbiota of SD and WKY rats significantly differed in the relative abundance of the KEGG functions Lipid Metabolism (SD 2.80 ± 0.01; WKY 2.75 ± 0.01; % ± SEM; FDR < 0.01) and Energy Metabolism (SD 5.39 ± 0.04; WKY 5.58 ± 0.04; % ± SEM; FDR = 0.02). No significant differences were detected between stressed and control rats, regardless of rat strain. Hierarchical clustering analysis of predicted metagenome KEGG functions showed a good separation between SD and WKY rats (Fig. 3).

Caecal short chain fatty acid (SCFA) concentrations were clearly differentiated between the two rat strains. Levels of isobutyric (*P* = 0.0003) and isovaleric (*P* = 8.93e-05) acids were higher in WKY than SD rats, whereas levels of butyric (*P* = 0.0045) and succinic (*P* = 0.0026) acids were lower. However, there was no significant difference in SCFA concentrations between stressed *vs* control rats within strains (Supplementary Fig. S4). There were no significant differences in the other SCFA measured (acetic, propionic, valeric, caproic, lactic and formic acids), either between strains or between stressed *vs* control rats within strains.

### Associations between microbe and metabolite relative abundances

**Figure 4:**
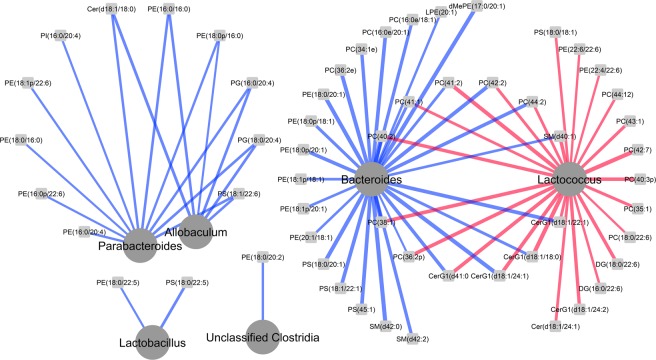
Network analysis showing canonical correlations between taxa and brain lipids for combined WKY and SD rat data. Positive correlations are shown in red and negative correlations in blue.

Canonical correlation analysis of all metabolites across treatment groups showed that relative abundances of *Bacteroides* across all rats was negatively correlated (association score < −0.65) with a wide range of phospholipids and phospholipid metabolites including several phosphtatidylcholines, phosphtatidylethanolamines, ceramides, and sphingolipids (Fig. 4). However, *Lactococcus* showed the reverse propensity, where relative proportions correlated positively (association score > 0.6) with a range of the same metabolites and lipids in the brain.

## Discussion

The results of this study show that WKY and SD rats responded differently to acute stress with respect to observed microbiome and metabolome changes. The main new finding from this study is the strong stress response exhibited in acutely challenged WKY rats and concomitant alteration in brain lipid profile. In contrast, the SD stress response was characterized by changes in plasma lipids. This reveals an important relationship between brain and peripheral physiological responses together with changes in the functional gut microbiome readouts. Associated with different behaviour in the stress challenges, our results show that SD and WKY rats also exhibit different physiological stress responses, as characterized by distinct brain or plasma metabolite profiles in the host, and caecal microbiota profiles. Indeed, exposure to acute stress had little impact on the caecal microbiome in SD rats, but led to a consistent shift in the microbial community in WKY rats as shown by random forest classification.

As expected based on previous literature^22,23,32^, anxiety-prone WKY rats were more disposed to depression-like and anxiety-like behaviours than their normo-anxious SD counterparts, as determined by the FST and OFT respectively. As expected, corticosterone elevations confirmed the expected impact of the acute stressor (FST) on HPA axis activation. These results support the findings of previous studies where WKY rats had expressed increased depression-like behaviour compared with SD rats during the FST^3,22,33,34^, and increased anxiety-like behaviour in the OFT compared to SD rats^35,36^. However, in contrast to the OFT and FST, the NOR test is used to evaluate memory. Our results show that SD rats displayed similar interest in the novel object to WKY rats, suggesting there was no difference between strains in terms of memory and learning.

*Limitations:* We acknowledge the need to be somewhat cautious in interpreting the results as a consequence of the behavioural procedures. Exposure to behavioural testing and the FST did produce a functional change in the microbiome over a 10-day period. These were relatively minor effects on microbiome composition affecting low abundance taxa. Nevertheless, changes in minor members of the microbiome can still influence physiological outcomes. For example, the presence of methanogens, which rarely exceed 1% of the microbiome in humans, can have an important effect on carbohydrate fermentation by other members of the microbiome through removal of H_2_^37^.

Because this study was focussed on rat strain comparisons rather than sex differences, we used only one gender of animal. Male rats were used because male WKY can show a greater susceptibility to acute stress than females in the OFT^38^. WKY females can be more immobile in the FST than males^39^, but this is not always the case^33^. While a more expansive sex-difference analysis would be informative it was beyond the scope of this study. Potential differences between male and female rats in their brain neurochemistry and microbiota response to stress will be the focus of future studies.

Whole brain was used to obtain a broad picture of whether brain chemistry was altered, which has been useful in initial studies^40,41^. This allowed us to examine the overall macro-physiological changes in brain lipids and polar metabolites in response to stress and whether these differ broadly between rat strains. Future studies will look to measure changes in specific regions of the brain to compare with that of whole brain and thus assess localised versus macro changes in brain metabolism.

Associated with an increased anxiety-like behaviour phenotype, WKY rats exhibited a consistent low level global alteration in their brain lipid profile in response to an acute stressor. This has not been reported previously and was characterized by lower levels of structural neuronal membrane lipids and phospholipids, and increased sphingomyelin. An apparent alteration in prominent stress-associated brain lipid profiles absent in SD rats may implicate increased brain lipid metabolism as characteristic of a stress-prone physiological response specific to WKY rats.

In contrast with the response seen in WKY rats, in SD rats (considered to represent a “normal” stress response strain of rat) plasma triglycerides and free fatty acids were elevated in response to stress. However, no stress-related changes in plasma lipids were observed in WKY rats. SD rats have been previously been shown to have increased plasma total lipid levels in response to acute and chronic stress^42^. In human clinical studies, acute stress has been associated with norepinephrine (NE)-induced lipolysis resulting in plasma lipid changes^43^. Increases in circulating NE can induce lipolysis and release free fatty acids into the bloodstream, which serve as a substrate for the re-synthesis of triglycerides and production of low-density lipoprotein cholesterol in the liver to fuel the “flight or fight” response^43,44^. Our results may therefore reflect differences in hormonal changes in response to stress between these rat strains, resulting in mobilization of fatty acids from the brain (WKY rats), rather than adipose tissue (SD rats).

There were no differing polar metabolites in the brain between stressed and control rats in either strain. There was, however, some evidence (albeit weak) that glutamine and tyrosine were elevated in the brain of stressed SD rats, suggesting that these contribute to a normal neurochemical stress response. Not surprisingly, dysfunction of glutamatergic neurotransmission is increasingly considered to be a core feature of stress-related mental illnesses^45^.

Plasma levels of glutamic acid, GABA and 3-methoxytyrosine were lower in WKY rats in response to stress, despite no change in the brain. The failure to increase plasma and brain tyrosine levels by WKY rats in response to stress supports the hypothesis that reduced arousal and behavioural responsivity in WKY rats may be related to deficient brain noradrenergic reactivity, thus contributing to their increased susceptibility to stress^46^.

Together, our data show that these specific strain-dependent changes in brain and plasma metabolites in response to stress, particularly brain lipids, may reflect pre-existing metabolomics and microbiota differences which would prime the baseline to differential responses to an acute stressor. Strain-dependent changes in neurotransmitter and neurotrophin levels in response to chronic stress are also thought to predispose these animals to a depressive-like phenotype compared to SD rats^32^.

As expected, we found that the caecal microbial communities were clearly delineated between SD and WKY rats based on the unweighted Unifrac analysis, despite sharing many of the same taxa. Stress was also found to impact the function of SD and WKY microbiomes differently. In SD rats, exposure to stress-induced behaviour testing had only minor effects on microbiome composition, with changes mainly occurring among low relative abundance Proteobacteria, which includes *Desulfovibrio*. Sulfate metabolism by *Desulfovibrio* generates H_2_S, a signalling molecule that regulates several physiological responses including inflammation and neuronal activity^47^. While it is unknown if H_2_S generated by *Desulfovibrio* plays a significant role in modulating brain function, *Desulfovibrio* abundance has been shown to be associated with the increasing severity of autism in children^48^. In WKY rats, however, exposure to stress shifted the microbiome in a small but consistent manner, with changes mainly amongst the Firmicutes, such as *Lactococcus* and *Lactobacillus* (members of the *Lactobacillales*), and *Roseburia* and *Ruminococcus* (members of the *Clostridiales*), all of which are lactate, succinate or SCFA producers. These bacteria all play a role in carbohydrate fermentation, which is a core activity of the microbiome in determining energy and carbon balance in the gut^49^.

This study also demonstrates that certain bacterial taxa and brain lipids have parallel patterns of relative abundance that are rat strain-specific. In particular, *Lactococcus* relative abundances were highly correlated with the relative proportions of many brain lipids. Notably, *Lactococcus* was the only genus showing significant change in response to acute stress in WKY rats. A rapidly growing body of evidence, particularly in preclinical models of anxiety/stress, depression and IBS, has shown a clear link between the gut microbiota and brain and behaviour via communication along the microbiota-gut-brain axis^1,9,50–59^. Another clear pattern that emerged was the high negative correlation between the relatively abundances of *Bacteroides* and *Parabacteroides* and a wide range of brain lipids and lipid metabolites. While the physiological significance of this link is unclear, decreased *Bacteroides* has been associated with higher clinical depression and anxiety symptoms in IBS patients^60^. In contrast, *Lactococcus* positively correlated with 12 of the lipids that decreased with increasing *Bacteroides* proportions, raising the possibility that these two genera may have indirect roles in brain lipid metabolism. Although the role of autochthonous *Lactococcus* in modulating brain function is unexplored, *Lactococcus lactis* and other members of the *Lactobacillales* such as *Streptococcus thermophilus* and *Lactobacillus bulgaricus* have been shown to improve mood in healthy human subjects when consumed as a probiotic milk drink^61^.

While exposure to stress did not alter concentrations of SCFA and other measured acids in either rat strain, the microbiome functional outputs observed in WKY rats suggest that energy and lipid metabolism may play a role in modulating gut-brain signalling in this rat strain. Other researchers have shown that SCFAs produced by the gut microbiota behave as signalling molecules with downstream neurochemical effects via the gut-brain axis^1,47^. In this case, whole body SCFA turnover analysis may provide more insight^62^. While SCFA levels did not differ in response to stress within strains, it was interesting to note that iso-butyric and iso-valeric concentrations were higher in stressed WKY rats compared to stressed SD rats. Our findings are in line with increased levels of isobutyric and isovaleric acids observed in patients with gastrointestinal diseases shown to have downstream neurological effects, such as celiac disease^63^ and IBD^64^, as well as autism spectrum disorder^65^. Increased levels of isovaleric acid in stools were also found to correlate with human depression^66^.

A key finding in our study has been a strong trend toward altered lipid metabolism between SD and WKY rats, which may, at least in part, explain differences in behaviour between the two rat strains. Supporting this, we also found that the greatest predicted functional difference between the SD and WKY microbiomes was the relative abundance of bacterial genes involved in lipid and energy metabolism. The microbiome has already been shown to alter host lipid metabolism through several mechanisms, including SCFA signalling-induced modification of metabolism and insulin sensitivity^67^, and microbial bile acid metabolism^68^. It is therefore conceivable that direct microbial metabolism of lipids may also have an influence on the host lipid metabolism. Overall, our results suggest that the acute stress response in WKY rats involves altered host-microbiota interactions. It is important to clarify that not all stress is detrimental. A functional stress response is necessary to avert danger and is critical during brain development.

## Conclusion

This study is the first to provide comprehensive analyses of the plasma and brain metabolomes, and caecal microbiome composition, in response to stress in two rat strains; WKY and SD rats. A key finding from our study has been the identification of differential physiological responses to acute stress, particularly with respect to lipid metabolism, where SD rats had increased levels of plasma lipids while WKY rats had altered brain lipid profiles. Whether brain or gut physiological changes are driving the GBA stress response remains to be determined. Intriguingly, this suggests that under the experimental conditions we used, signalling from the gut had more impact on the physiological response to acute stress in WKY rats than it did in SD rats. We have presented examples of features differentiated in response to stress, as well as between rat strains, that suggest lipid profiles are a potential biomarker for studying the effect of foods, drugs or behaviour on stress and anxiety, in addition to supporting the selection of appropriate rodent models. By comparing a stress prone strain with a normo-anxious strain we have been able to explore what a healthy stress response may entail in terms of the microbiota and metabolome.

## Methods

### Animals

This study was reviewed and approved (application 13501) by the AgResearch Grasslands Animal Ethics Committee (Palmerston North, New Zealand) according to the Animal Welfare Act (1999). Twenty four male Sprague Dawley (SD) rats and twenty four male Wistar Kyoto (WKY) rats were received from the Animal Resources Centre (Canning Vale, WA, Australia) at 8 weeks of age. SD rats are widely used as controls for WKY rats^3,32,33^. Animals were allowed to acclimatize to the facility and were handled daily for 1 week before being used in experiments. Animals were housed individually at a constant temperature of 21 °C and maintained under a 12/12 h light/dark schedule (lights on at 7:00 am). At 10 weeks of age, rats of each strain were randomly assigned to one of two treatment groups (n = 12 per group) where one group was subject to behavioural testing while the other group was not tested (Supplementary Fig. S5). All rats were fed an adult maintenance diet (AIN-93M; OpenStandard Rodent Diet, Research Diets, Inc. New Brunswick, NJ, USA) and water, provided *ad libitum*. Animals were monitored three times weekly for weight, food intake, and General Health Score (1–5; NZ Animal Health Care Standard). WKY rats are smaller (~260 g) than SD (~450 g) and eat 18 and 28 g per day, respectively^69^. Behaviourally tested animals were sacrificed immediately following completion of FST experiments. At the end of the study the rats were euthanized using carbon dioxide inhalation overdose.

### Behavioural testing

The experiments were performed during the light phase (between 8:00 am and 12:00 pm). Half the rats underwent behavioural testing which acted as a stressor using the OFT, NOR and FST. Rats underwent the OFT (day 21) as described by McKernan *et al*.^54^, and NOR testing (day 24) as described by Pusceddu *et al*.^70^ which included habituation (day 22) and pre-testing (day 23). Behaviour for the OFT and NOR was recorded and analysed using EthoVision XT 10 (Noldus, Wageningen, The Netherlands). Blood sampling via tail tipping was undertaken immediately prior to testing on Day 2 of the FST (day 32) which was carried out as previously described^71^. Behaviour (time spent swimming, immobile or climbing) and the number of faecal pellets produced were manually recorded by an observer blinded to the treatment groups. Rats were removed from the testing room immediately after completion of the forced swim test and euthanized.

### Sample collection

Blood samples were taken from behaviourally tested rats just prior to the FST (8–10 am), in which our sampling window corresponded with low basal resting cortisosterone levels to provide a baseline measure^72–74^, as well as immediately post-mortem. Plasma from tail tipped blood samples was prepared by filling two haematocrit capillary tubes (approximately 3/4 full), sealing the dry end with critoseal® (Leica Microsystems GmbH, Wetzlar, Germany) and centrifuged (microhaematocrit centrifugation (microhematocrit centrifuge type 346, MSE Scientific Instruments, UK) for 5 minutes at 2500 × *g*. Haematocrit tubes were snapped, and plasma expelled into labelled tubes. Tissue samples (brain (whole) and gastrointestinal – ileum, jejunum, caecum and colon and their contents) were collected immediately after euthanasia. Intestinal contents were separated from their respective tissue while the tissue was washed in cold saline and treated with RNA*later*^®^ (Invitrogen) as previously described^75^. Post-mortem blood samples were removed by cardiac puncture using a 21-gauge needle and syringe pre-rinsed with EDTA, centrifuged at 2000 × *g* for 5 minutes at r.t. and the plasma removed. All samples were snap frozen in liquid nitrogen and stored at −80 °C until use.

### Corticosterone ELISA

Plasma corticosterone levels were measured using a commercial kit (Corticosterone ELISA kit, Enzo Life Sciences, Farmingdale, NY, USA) as per the manufacturer’s instructions, and the concentration of each sample was extrapolated from a standard curve. Sensitivity of the assay was 27.0 pg/mL (range 32–20,000 pg/mL).

### Caecal microbiota

DNA was extracted from caecal contents using the NucleoSpin Soil kit (Macherey-Nagel GmbH, Düren, Germany) according to a previously described method^76^. The V3-V4 region of the bacterial 16S rRNA gene was amplified using 16S dual-indexed primers^77^. Amplicons were sequenced at NZGL Ltd. (Palmerston North, New Zealand) using the MiSeq with 2X 250 base PE chemistry. Paired end sequences were joined using the join_paired_ends.py script and sequences were quality filtered (q30) using the Qiime 1.8 pipeline^78^. The resulting sequences were chimera checked using the USEARCH method against the Greengenes alignment (release GG_13_8), following which chimeric sequences were removed from subsequent analyses. Sequences showing 97% or greater similarity were clustered into operational taxonomic units (OTUs) using the UCLUST method and representative sequences were assigned taxonomies using the RDP classifier. Common taxa across the dataset were determined using the compute_core_microbiome.py script in Qiime. Random forest classification was performed using the randomForest package for R^79^. Predicted metagenome functional classifications were obtained using PICRUSt^80^ via the online Galaxy server. Sequence reads are publicly assessible from the NCBI Sequence Read Archive under the accession code PRJNA562079 (https://www.ncbi.nlm.nih.gov/bioproject/PRJNA562079).

### Short chain fatty acid (SCFA) analysis

Caecal contents (200 mg) were prepared in a two-step procedure as previously described^81,82^. In the first step, SCFA were extracted into an aqueous solution for Gas Chromatography – Flame Ionization Detector (GC-FID) analysis of acetic, butyric, propionic, valeric, *iso*-valeric, *iso*-butyric and caproic acids. In the second step, an ether extraction was performed followed by derivatisation with N-tert-Butyldimethylsilyl-N-methyltrifluoroacetamide (MTBSTFA) for GC analysis of lactic, formic and succinic acids. Briefly samples were homogenized in PBS and centrifuged at 21,000 × *g* for 10 min at 4 °C. The supernatant (700 µL) was transferred to a fresh tube and 78 µL of internal standard added. Samples were stored at −20 °C overnight, and defrosted the following morning in a RT water bath and centrifuged at 21,000 × *g* for 10 min at 4 °C. Supernatants (600 µL) were transferred to GC vials and underwent GC-FID analysis. For GC analysis, 200 µL of supernatant was combined with 100 µL 37% HCl, 5 µL resazurin dye and 800 µL of ether, shaken vigorously, left to settle for 1 min and the top ether layer transferred to a fresh tube. A further 800 µL of ether was added to the aqueous extract; samples were treated as before and the top ether layer removed. The pooled ether extract (800 µL) was combined with 100 µL of derivatizing agent (MTBSTFA) in GC vials, heated at 80 °C for 20 min, incubated at RT for 48 h to complete derivatization, then run on a GC mass spectrometer.

### Metabolomic analyses

Extractions were performed using the method of Armirotti, *et al*.^83^ which was capable of generating extracts for both the HILIC and lipid analyses from a single aliquot of plasma or brain tissue. Briefly, 200 µL plasma or 50 mg of homogenized brain tissue was extracted by bi-phasic liquid–liquid extraction using a mixture of water/methanol/chloroform/heptane. The upper (aqueous) phase removed (200 µL), dried under N_2_ and reconstituted in 200 µL of 50:50 acetonitrile:water containing 10 µg/mL d_2_-tyrosine as an internal standard for HILIC-MS analysis. Likewise, 200 µL of the organic (lower) phase was dried under N_2_ and reconstituted in 200 µL of 2:1 chloroform:methanol (v/v) containing d31-PE internal standard at 10 µg/ml. To verify and/or maintain data quality within each mode, a QC sample (comprising a pooled extract of a sub-set of samples for brain analyses, and a bovine plasma sample for plasma analyses) was also injected once every 10 samples. Plasma and brain extracts were analysed through HILIC and lipid LC-MS streams using both positive and negative ionization modes as previously described^84,85^.

Metabolites eluting between 3–18 minutes for the HILIC analysis and between 1–11 min for the lipidomics analysis were extracted from the LC-MS data by converting the data files to mzXML file format (ProteoWizard^TM^) and performing peak detection, alignment and grouping using XCMS. The resultant peak intensity table was subjected to an in-house linear run-order correction normalization and isotope/adduct annotation using respective R based software. Data corresponding to isotopes and background noise were removed from the final data matrix. Feature annotation for HILIC-MS was performed by matching peaks against an in-house library of authentic standards run under identical conditions. Where hits were unsuccessful, statistically significant features were searched against public domain databases HMDB and METLIN (mass tolerance of 5 ppm). Lipid LC-MS annotations were performed by matching the XCMS generated data matrix to lipids identified in the samples by MS^2^ spectral matching using LipidSearch^TM^ software (Thermo). Metabolomics data have been deposited to the EMBL-EBI MetaboLights database with the identifier MTBLS1192 (https://www.ebi.ac.uk/metabolights/MTBLS1192).

### Statistical analysis

Behavioural and corticosterone data were analysed by analysis of variance (ANOVA) using GenStat^®^ 18 (VSN International Ltd., UK). For microbiota and SCFA data, differences in mean proportions of taxa at the family level were analysed using two factor non-parametric permutation ANOVA (2000 permutations per test) as implemented in the RVAideMemoire package^86^ in R 3.0.2^87^, with the factors being rat strain and whether the rats were subjected to behavioural tests or not. P values < 0.05 were considered significant.

PICRUSt analysis of the predicted bacterial metagenome was performed using the Galaxy web app (http://huttenhower.sph.harvard.edu/galaxy) using default parameters; operational taxonomic units (OTU) were normalised by 16S rRNA gene copy numbers and KEGG Ortholog abundances were predicted based on OTU abundances^80^.

Hierarchical clustering analysis of predicted metagenome KEGG functions was performed using a Euclidean distance matrix and complete hierarchical clustering as implemented by the heatmap.2 function from the gplots R package.

Canonical correlation analysis to assess possible associations between microbe and metabolite relative abundances was performed using the splsda function in the mixOmics package for R^88^.

Metabolomics data analysis was performed using MetaboAnalyst v3.0^89^ and the statistical software package SIMCA (v14.0). Data from the two ionization modes for each of the chromatographic analyses (HILIC and lipid) were combined for statistical analysis, log2 transformed and auto-scaled. Univariate and multivariate data analyses were conducted and principal component analysis (PCA) used for dataset overview and to identify potential run order effects. Fold Change (FC) and t-test analysis of the strains were performed and a false discovery rate (FDR) correction utilized to reduce the risk of false positives. MS features with FDR < 0.1 for HILIC and FDR < 0.05 for lipid analysis were considered to differ significantly between strains.

Correlation between microbial and metabolite relative abundances were analysed by sparse Partial Least Squares (sPLS) regression analysis using the mixOmics package for R^90^.

## Supplementary information

Supplementary Information
