# Supplementary material for: Metabolome and microbiome profiling of a stress-sensitive rat model of gut-brain axis dysfunction
Source: Sci Rep. 2019 Oct 1;9:14026. doi: 10.1038/s41598-019-50593-3 (PMC6773725; doi:10.1038/s41598-019-50593-3)
Supplement: Supplementary file 1 — Supplementary Information [file 41598_2019_50593_MOESM1_ESM.pdf]

Supplementary Information

Metabolome and microbiome profiling of a stress-sensitive rat model of gut-brain axis dysfunction

Shalome A. Bassett, Wayne Young, Karl Fraser, Julie E. Dalziel, Jim Webster, Leigh Ryan, Patrick Fitzgerald, Catherine Stanton, Timothy G. Dinan, John F. Cryan, Gerard Clarke, Niall Hyland, and Nicole C. Roy

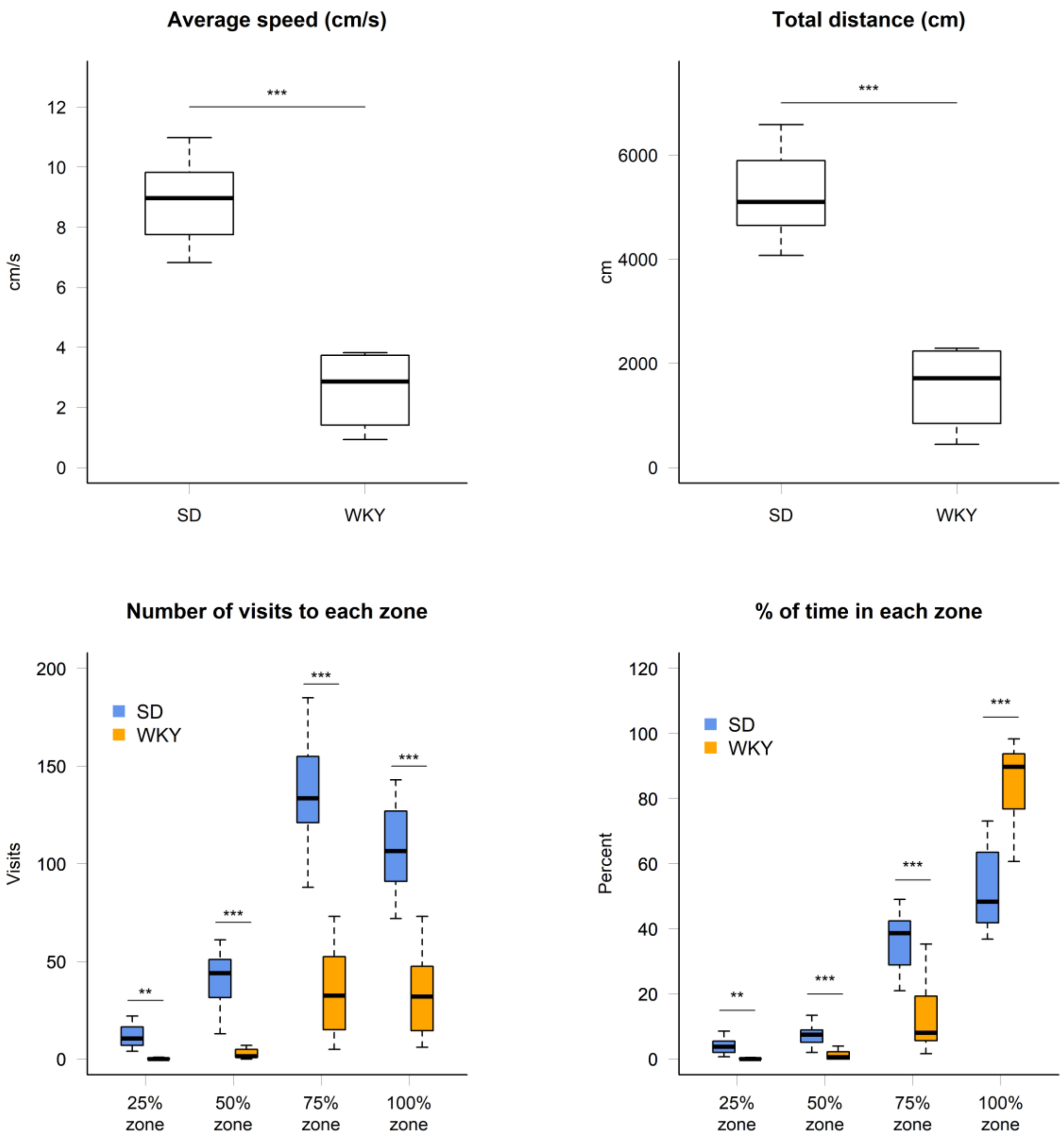

Fig. S1: Open field test results. SD and WKY rats differed across all measured aspects (P<0.01 and P<0.001, respectively). The mean and SEM are shown. SD = Sprague Dawley; WKY = Wistar Kyoto rats. The arena was divided into four zones where the 25% zone refers to the inner zone and the 100% zone refers to the outer zone, respectively.

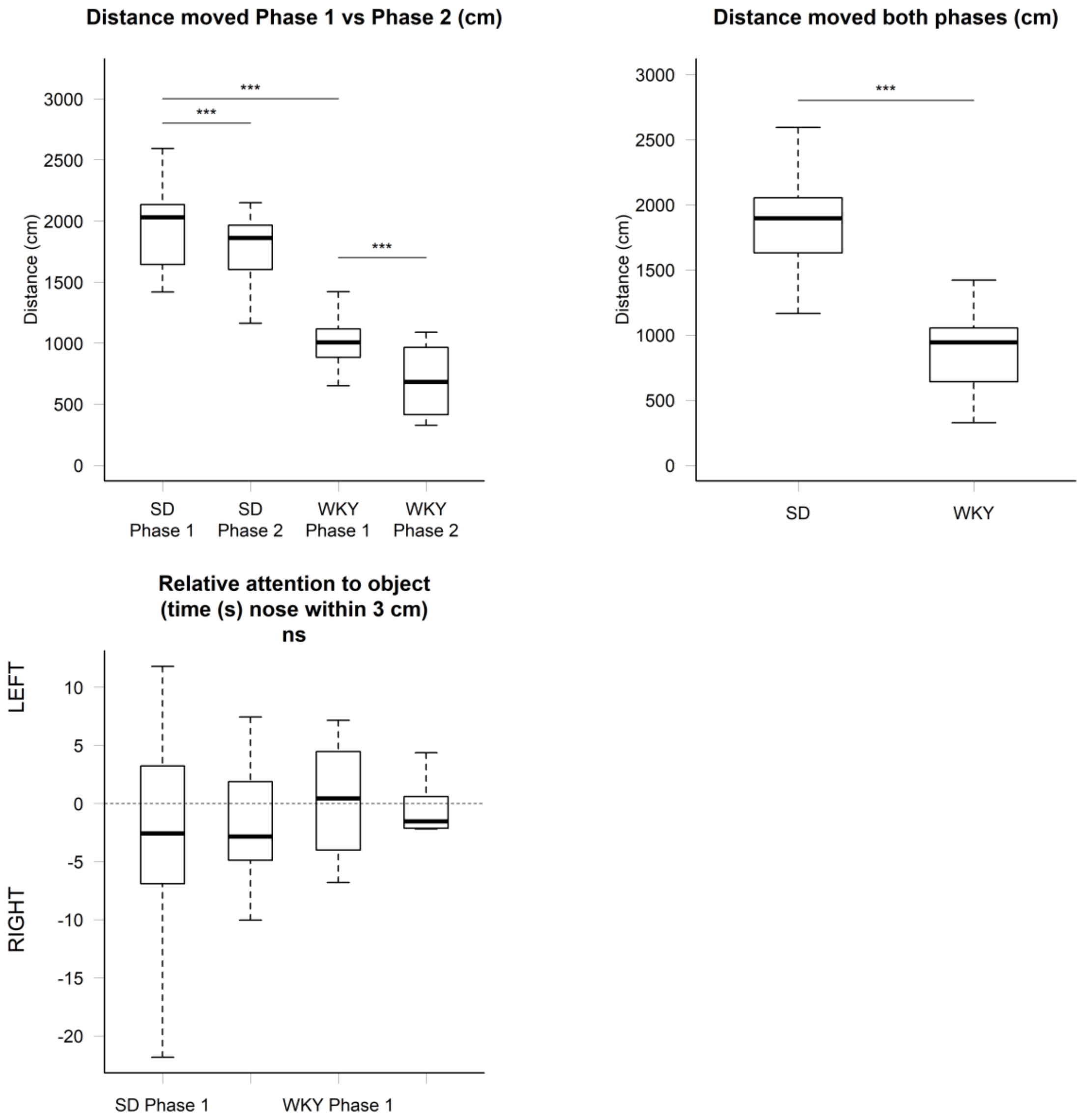

Fig. S2: Novel object recognition test results showing (A) the exploration time (i.e. the amount of time spent exploring either the old (familiar) or novel object) and (B) the Discrimination Index where a positive score indicates more time spent with the novel object. While both strains showed more interest in the novel object, there was no significant difference between strains. The mean and SEM are shown. SD = Sprague Dawley; WKY = Wistar Kyoto rats.

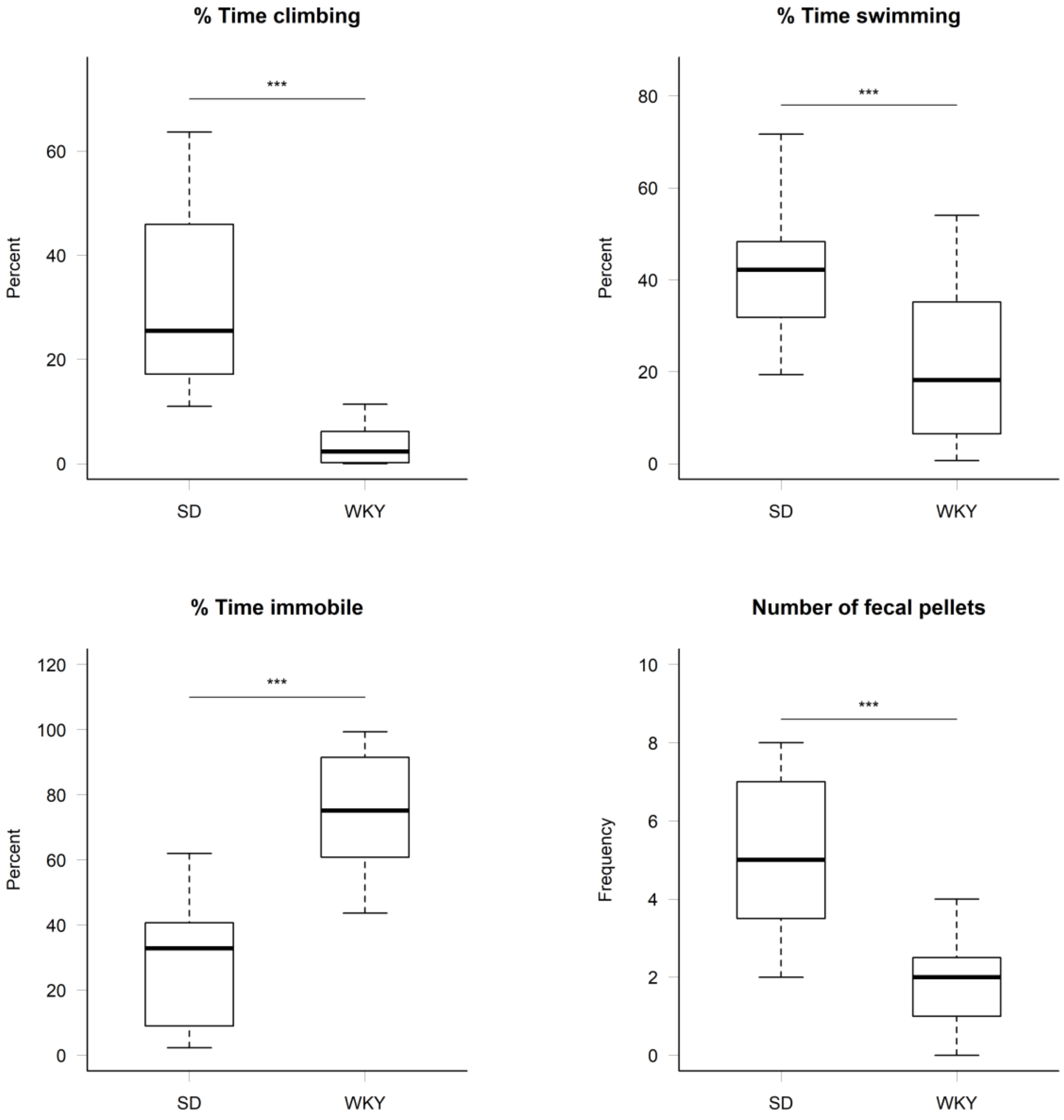

Fig. S3: Forced swim test results showing the percentage of time spent climbing, swimming or immobile. The number of faecal pellets produced were also measured. The mean and SEM are shown. SD = Sprague Dawley; WKY = Wistar Kyoto rats.

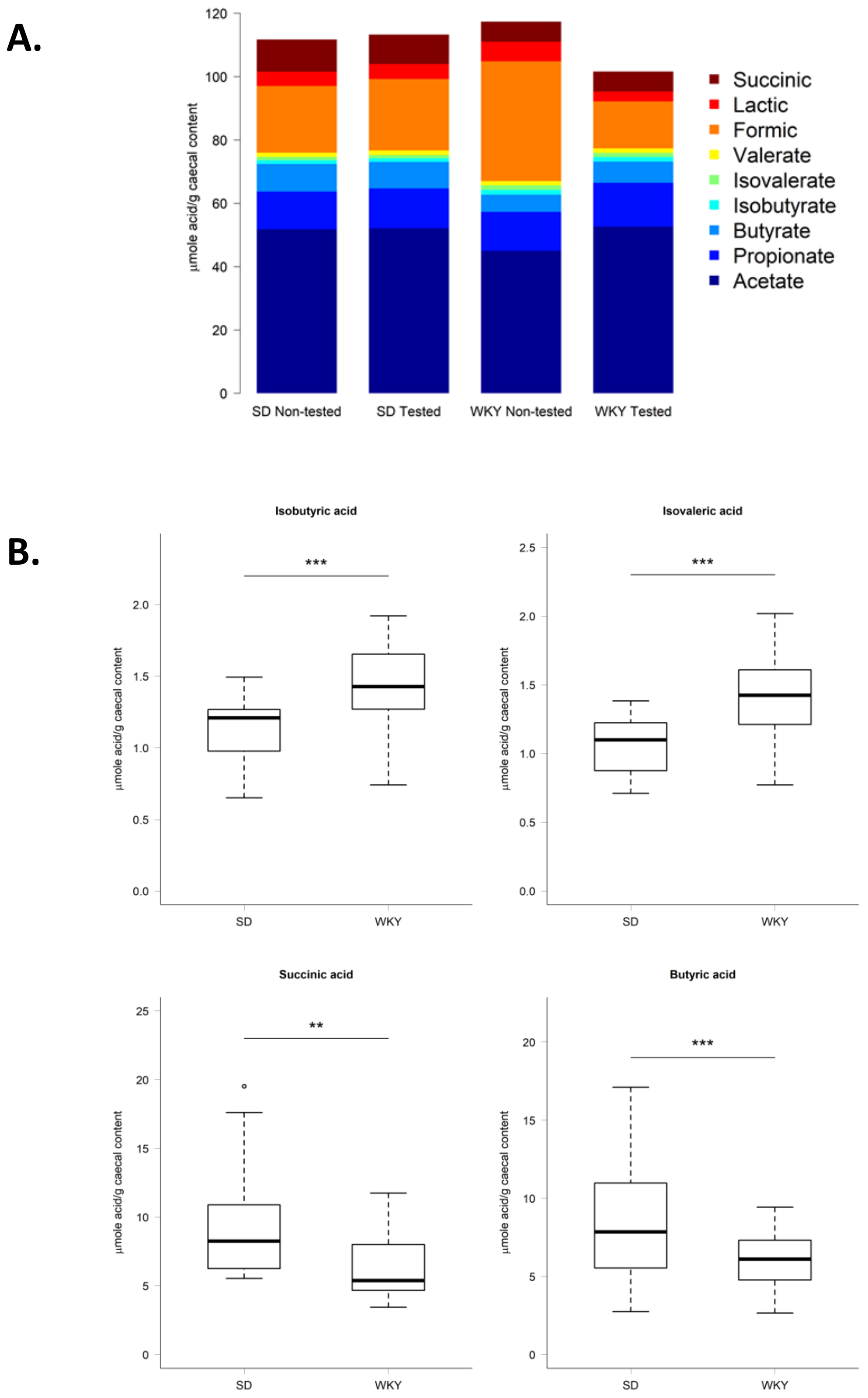

Fig. S4: (A) Mean concentration of caecal short chain fatty acids (SCFA); (B) Levels of isobutyric, isovaleric, butyric and succinic acids were significantly different between strains but not between tested *vs* untested rats within strains.

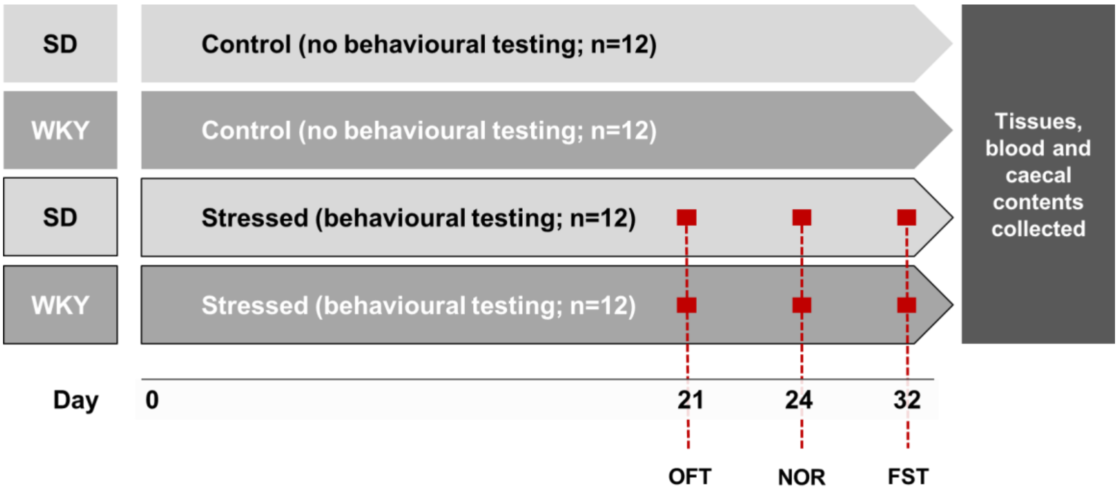

Fig. S5: Study design. Ten week old male Sprague Dawley (SD) and Wistar Kyoto (WKY) rats were individually caged and fed a standard rodent AIN-93M diet for 32 days. Half the rats underwent behavioural testing which acted as a stressor using the Open Field Test (OFT), Novel Object Recognition (NOR) and Forced Swim Test (FST) on Days 21, 24 and 32, respectively and were immediately euthanized after completing the FST. Blood samples were taken from behaviourally tested rats just prior to the FST (8-10 am) as well as immediately post-mortem. Tissue and caecal samples were also collected for further analyses.

Table S1: Mean corticosterone concentrations

Group	Mean Corticosterone (pg/mL)	SEM
**SD Control terminal**	6287.51	1042.628
**SD Pre-FST**	6762.10[a]	810.9855
**SD Stressed terminal**	19928.66[b]	1899.201
**WKY Control terminal**	7467.42	940.0187
**WKY Pre-FST**	6863.04[a]	556.9703
**WKY Stressed terminal**	18309.09[b]	1359.153

[a] Significant difference (*P*<0.001) between pre-FST and terminal corticosterone levels in stressed rats (both strains)

[b] Significant difference (*P*<0.001) between stressed and control rats (both strains)

Table S2: Random forest classification of microbiome from SD and WKY rats subjected to acute stress or non-stressed controls.

Group	Classified as:		Class error
	Non-stressed Control	Stressed	
SD non-stressed	3	9	75.0%
SD stressed	5	7	41.7%
WKY non-stressed	10	2	16.7%
WKY stressed	2	10	16.7%

Table S3: Plasma lipid changes in response to stress. The log2 fold change (log_2FC) in response to stress for each rat strain is shown. Lipids highlighted in orange were consistently different between rat strains.

SD rats	log_2FC SD-stressed/SD-control	WKY rats	log_2FC WKY-stressed/WKY-control
TG(28:1/16:0/18:1)+NH4	1.28	TG(16:0/14:0/16:1)+NH4	0.69
TG(16:0/18:1/24:0)+NH4	1.20	TG(16:0/14:0/18:1)+NH4	0.64
TG(18:0/18:2/24:0)+NH4	1.19	TG(16:0/16:1/16:1)+NH4	0.63
TG(16:0/18:1/23:0)+NH4	1.18	TG(18:0/18:2/24:0)+NH4	0.56
TG(18:1/18:1/23:0)+NH4	1.18	TG(30:1/18:1/18:1)+NH4	0.54
TG(30:1/16:0/18:2)+NH4	1.09	TG(16:0/14:1/16:1)+NH4	0.47
TG(16:0/18:2/23:0)+NH4_2	1.08	TG(15:0/16:0/18:1)+NH4	0.46
TG(26:1/18:1/18:2)+NH4	1.08	TG(16:0/18:1/19:0)+NH4	0.45
TG(30:1/18:1/18:1)+NH4	1.06	TG(15:0/16:1/18:2)+NH4	0.44
TG(26:0/16:0/16:0)+NH4	1.05	TG(18:0/16:0/18:1)+NH4	0.42
TG(16:0/16:0/23:0)+NH4	1.04	TG(16:0/16:0/18:1)+NH4	0.40
TG(26:1/18:2/24:2)+NH4	0.95	TG(15:0/16:0/16:1)+NH4	0.40
TG(18:1/18:2/23:0)+NH4	0.94	TG(16:1/16:1/18:1)+NH4	0.39
TG(16:0/18:1/19:0)+NH4	0.92	TG(18:1/18:2/24:0)+NH4	0.38
TG(18:1/18:2/24:1)+NH4	0.90	TG(16:0/16:1/18:1)+NH4	0.38
TG(16:0/18:1/24:1)+NH4	0.86	TG(15:0/16:0/18:2)+NH4	0.37
TG(18:1/18:2/24:0)+NH4	0.78	TG(16:0/18:1/23:0)+NH4	0.37
TG(18:0/18:0/18:1)+NH4	0.78	TG(26:0/18:1/18:2)+NH4	0.35
TG(18:0/16:0/18:0)+NH4	0.75	TG(16:0/14:0/16:0)+NH4	0.35
TG(16:0/18:2/24:1)+NH4	0.74	TG(16:1/14:0/18:2)+NH4	0.34
TG(18:0/16:0/16:0)+NH4	0.71	TG(26:1/18:2/24:2)+NH4	0.33
TG(26:0/18:1/18:2)+NH4	0.70	TG(26:1/18:1/18:2)+NH4	0.32
TG(15:0/16:0/18:1)+NH4	0.68	TG(16:0/18:1/24:0)+NH4	0.30
TG(25:0/18:2/18:2)+NH4	0.68	TG(18:0/18:0/18:1)+NH4	0.30
TG(16:0/16:0/16:0)+NH4	0.66	TG(16:0/16:0/16:0)+NH4	0.30
TG(17:0/18:1/18:1)+NH4	0.61	TG(16:0/18:1/24:1)+NH4	0.30
TG(18:1/18:2/22:1)+NH4	0.59	TG(16:0/18:2/23:0)+NH4_2	0.27
TG(15:0/16:0/16:1)+NH4	0.58	TG(16:0/17:1/18:1)+NH4	0.27
TG(16:1/18:2/20:5)+NH4_1	0.57	TG(16:0/14:0/20:4)+NH4	0.25
TG(18:0/16:0/18:1)+NH4	0.57	TG(26:0/16:0/16:0)+NH4	0.24
TG(14:0/18:2/20:4)+NH4	0.56	TG(18:0/16:0/16:0)+NH4	0.24
TG(16:0/14:0/16:0)+NH4	0.55	TG(17:0/18:1/18:1)+NH4	0.23
TG(18:3/18:2/18:2)+NH4	0.55	TG(28:1/16:0/18:1)+NH4	0.23
TG(16:0/14:0/16:1)+NH4	0.53	TG(30:1/16:0/18:2)+NH4	0.21
TG(15:0/16:0/18:2)+NH4	0.50	TG(12:0/18:2/18:2)+NH4	0.20
TG(16:0/14:0/18:1)+NH4	0.50	TG(18:1/18:2/23:0)+NH4	0.19
TG(16:0/16:0/18:1)+NH4	0.48	TG(16:0/18:1/21:0)+NH4	0.19
TG(15:0/16:1/18:2)+NH4	0.48	TG(18:1/18:1/23:0)+NH4	0.19
TG(18:1/18:2/18:3)+NH4	0.47	TG(16:0/16:0/23:0)+NH4	0.18

TG(15:0/18:2/18:2)+NH4	0.47	TG(18:1/18:2/24:1)+NH4	0.18
TG(16:0/16:1/16:1)+NH4	0.44	TG(25:0/18:2/18:2)+NH4	0.18
TG(12:0/18:2/18:2)+NH4	0.42	TG(16:0/18:2/24:1)+NH4	0.17
TG(16:0/14:1/16:1)+NH4	0.41	TG(16:0/18:2/23:0)+NH4_1	0.16
TG(16:0/17:1/18:1)+NH4	0.41	TG(18:0/16:0/18:0)+NH4	0.15
TG(20:1/18:1/18:1)+NH4	0.40	TG(16:1/16:1/18:3)+NH4	0.13
TG(16:0/18:1/20:1)+NH4	0.39	TG(18:1/18:2/22:1)+NH4	0.12
TG(18:0/18:2/18:3)+NH4_1	0.38	TG(17:0/18:1/18:2)+NH4	0.12
TG(16:0/18:2/18:3)+NH4	0.38	TG(16:0/18:1/18:1)+NH4	0.11
TG(18:2/17:1/18:2)+NH4	0.38	TG(20:1/18:1/18:1)+NH4	0.10
TG(22:1/18:2/18:2)+NH4	0.37	TG(16:0/18:1/20:1)+NH4	0.08
TG(20:3/18:2/18:2)+NH4	0.36	TG(18:1/18:1/18:1)+NH4	0.08
TG(16:1/16:1/18:3)+NH4	0.34	TG(14:0/18:2/20:4)+NH4	0.07
TG(17:0/18:1/18:2)+NH4	0.34	TG(15:0/18:1/18:2)+NH4	0.06
TG(16:0/16:1/18:1)+NH4	0.33	TG(15:0/18:2/18:2)+NH4	0.06
TG(18:0/18:2/18:3)+NH4_2	0.33	TG(20:1/18:1/18:2)+NH4	0.04
TG(18:1/17:1/18:2)+NH4	0.32	TG(20:1/18:2/18:2)+NH4	0.03
TG(15:0/18:1/18:2)+NH4	0.30	TG(16:0/18:2/21:0)+NH4	0.02
TG(16:1/14:0/18:2)+NH4	0.30	TG(16:0/18:1/18:2)+NH4	0.02
TG(16:0/14:0/20:4)+NH4	0.29	TG(18:1/17:1/18:2)+NH4	-0.01
TG(16:0/18:2/23:0)+NH4_1	0.27	TG(18:1/18:1/22:4)+NH4	-0.02
TG(16:1/16:1/18:1)+NH4	0.26	TG(16:0/16:1/22:6)+NH4	-0.03
TG(16:0/18:1/21:0)+NH4	0.24	TG(18:0/18:2/18:3)+NH4_2	-0.04
TG(16:0/16:1/22:6)+NH4	0.21	TG(18:1/18:1/18:2)+NH4	-0.06
TG(20:1/18:2/18:2)+NH4	0.18	TG(16:1/18:2/20:5)+NH4_2	-0.06
TG(16:0/16:0/20:4)+NH4	0.17	TG(20:3/18:2/18:2)+NH4	-0.06
TG(18:1/18:1/18:2)+NH4	0.17	TG(16:0/16:0/20:4)+NH4	-0.07
TG(20:1/18:1/18:2)+NH4	0.13	TG(18:2/17:1/18:2)+NH4	-0.09
TG(16:0/18:1/18:2)+NH4	0.10	TG(16:0/18:2/22:6)+NH4	-0.10
TG(16:0/18:1/18:1)+NH4	0.09	TG(22:1/18:2/18:2)+NH4	-0.10
TG(16:0/18:2/21:0)+NH4	0.07	TG(16:0/16:0/22:6)+NH4	-0.10
TG(18:1/18:1/22:4)+NH4	0.07	TG(18:0/18:2/18:3)+NH4_1	-0.11
TG(18:1/18:1/18:1)+NH4	0.06	TG(16:0/18:1/22:6)+NH4_2	-0.11
TG(18:2/18:2/22:6)+NH4_1	0.03	TG(18:1/18:2/22:4)+NH4	-0.11
TG(18:3/18:2/22:6)+NH4	0.02	TG(18:1/18:2/22:6)+NH4	-0.13
TG(16:0/16:0/22:6)+NH4	0.02	TG(16:0/18:2/18:3)+NH4	-0.14
TG(18:1/18:1/22:6)+NH4_2	0.00	TG(16:1/18:2/22:6)+NH4	-0.15
TG(18:1/18:1/22:6)+NH4_1	-0.01	TG(18:1/18:1/22:6)+NH4_1	-0.17
TG(16:1/18:2/22:6)+NH4	-0.02	TG(16:0/18:1/22:6)+NH4_1	-0.18
TG(16:0/18:1/22:6)+NH4_1	-0.06	TG(18:3/18:2/22:6)+NH4	-0.18
TG(18:2/18:2/22:6)+NH4_2	-0.06	TG(18:2/18:2/22:6)+NH4_1	-0.19
TG(16:0/18:1/22:6)+NH4_2	-0.20	TG(18:1/18:2/18:3)+NH4	-0.22
TG(16:0/18:2/22:6)+NH4	-0.20	TG(18:2/18:2/22:6)+NH4_2	-0.23
TG(16:1/18:2/20:5)+NH4_2	-0.23	TG(18:1/18:1/22:6)+NH4_2	-0.27
TG(18:1/18:2/22:4)+NH4	-0.27	TG(18:3/18:2/18:2)+NH4	-0.32
TG(18:1/18:2/22:6)+NH4	-0.33	TG(22:5/18:2/18:2)+NH4	-0.33
TG(22:5/18:2/18:2)+NH4	-0.80	TG(16:1/18:2/20:5)+NH4_1	-0.34
